# Pathology and Treatment of Diseases of the Skin for Practitioners and Students

**Published:** 1895-09

**Authors:** 


PATHOLOGY AND TREATMENT OF DISEASES OF THE SKIN FOR PRACTITIONERS AND STUDENTS. By DR. MORIZ KAPOSI, Professor of Dermatology and Syphilis and Chief of the Clinic and Division for Skin Diseases in the Vienna University. With eighty-four illustrations. Translation of the last German edition under the supervision of James C. Johnson, M. D. Price, muslin, $5.50; leather, $6.50. New York: William Wood & Co., Publishers. 1895.

This work will prove very serviceable, we feel sure, to the American practitioner and may be truly described as a popular text-book, the exponent of the treatment of diseases of the skin as practised at the Vienna School of Dermatology. The author's extensive experience and unrivaled opportunities for the clinical study of the various phases of cutaneous affections has enabled him to produce a book which is a credit to himself and an honor to the school of which he is the acknowledged head.

The illustrations are graphic and add to the usefulness as well as attractiveness of the volume.

The translation has been most careful and is in every respect a successful one. E. W.

